# Root Development of Bell Pepper (*Capsicum annuum* L.) as Affected by Water Salinity and Sink Strength

**DOI:** 10.3390/plants9010035

**Published:** 2019-12-25

**Authors:** Ran Erel, Thuc T. Le, Amram Eshel, Shabtai Cohen, Rivka Offenbach, Tobias Strijker, Ilana Shtein

**Affiliations:** 1 Gilat Research Center, Institute of Soil, Water and Environmental Sciences, Agricultural Research Organization, Negev 85-280, Israel; thethucbiotech@gmail.com; 2 Yair Station, Central and Northern Arava R&D Center, Hazeva 8681500, Israel; sab@inter.net.il (S.C.); Rivka@arava.co.il (R.O.); tovia@Arava.co.il (T.S.); 3 School of Plant Sciences and Food Security, Tel Aviv University, Tel Aviv 6997801, Israel; amrame@tauex.tau.ac.il; 4 Department of Oenology and Agriculture, Eastern R&D Center, Ariel 40700, Israel; ilanash@ariel.ac.il

**Keywords:** root to shoot ratio, fruit load, abiotic stress, root capacitance

## Abstract

Fruits are the dominant sinks for assimilates. At optimal conditions, assimilates supply can meet the demand of fruits and those of the vegetative organs; however, extreme circumstances such as strong sink strength or an environmental stress may disturb this fine balance. While most studies focus on aboveground parameters, information regarding root growth dynamics under variable sink strength are scarce. The objective of this study was to evaluate the effect of sink strength (represented by fruit load) and salinity on bell-pepper root development. Three levels of fruit load were combined with two salinity levels in plants grown in an aeroponic system. Root growth was determined both by root capacitance and destructive measurements. Salinity and sink strength significantly affected root, shoot and fruit growth dynamics. Root growth was less affected by fruit load. Salinity stress was negatively associated with shoot growth, but after an acclimation period, salinity enhanced root development. Additionally, this study shows for the first time that root capacitance is a valid approach for non-destructive measurement of root development in aeroponic systems. The good correlation measured by us (r^2^ 0.86) opens new opportunities for continuous root growth monitoring in aeroponic systems in the future.

## 1. Introduction

Successful plant growth and reproduction depend on adequate partitioning of the resources among roots, shoots and reproductive organs. Source and sink metabolism are tightly coordinated and large fluctuations and imbalances between supply and demand are avoided [1,2,3]. At the early stages of plant development, assimilates are partitioned between shoots and roots. On the other hand, at maturity the fruits are the dominant sinks for assimilates, and fruit growth adds a burden on root and shoot capacity for supply of nutrients, water and photosynthates. Interestingly, the sink strength of a developing fruit fluctuates during growth, and thus the fruit growth is often characterized by a sigmoid curve [4], implying complex intra-seasonal interaction with shoot and root development dynamics.

At the reproductive stage, fruits are the dominant sink and substantially modify shoot and root growth dynamics by competing for the assimilates produced by the leaves [5]. Various studies have examined the effect of fruit load on root development in different crops [6,7,8,9]. Villena et al. [9] studied peach root dynamics using minirhizotrons. They demonstrated that root growth continued throughout the season with lower rates during the active fruit growth phase. But, root growth rates increased immediately after harvest. Lenz, [10] found in apples that, while the portion of the fruits increased from 2% to 49% of the total biomass, the portion of roots decreased from 31% to 18%. It has also been shown that the nutrient uptake by non-fruiting trees is greater than by fruiting trees. This is due to the lower root biomass of fruiting trees, and because the uptake of mineral nutrients is in direct proportion to the total dry weight increase in the vegetative tree organs. However, in hydroponically grown bell peppers, fruit load did not influence the root dry matter percentage [11].

Abiotic stresses include salinity, drought and high temperature, impair growth and yield [12]. The harmful effect of salinity on plant growth includes the direct effects of ion toxicity and the indirect effects by reducing water potential. Salinity reduces the ability of plants to take up water, and consequently, leads to stomatal closure and reduction in growth rate and yield, along with a suite of metabolic changes similar to those caused by water stress [13,14,15].

The bell pepper (*Capsicum annuum* L.), is commonly cultivated and consumed all over the world. It is considered an important commercial and dietary vegetable crop grown mainly in greenhouses or net-houses. Pepper is considered a salt sensitive plant species [16]. In an arid environment, irrigation with saline water (EC 3.6 dSm^−1^) caused yield reduction of up to 50% compared with desalinated water [17,18,19]. To date, most studies have focused on aboveground parameters; detailed information regarding root characteristics and development under salinity is scarce. The main reason for the limited information on root development dynamics is the technical challenges involved in the determination of root biomass. Common methods for root growth measurement are, generally speaking, either laborious (e.g., “shovelomics” [20]), highly variable (e.g., the mini-rhizotron technique [21]) or require sophisticated and expensive equipment (e.g., MRI or microCT) [22]. An alternative approach for monitoring root development is the measurement of the root capacitance of plants grown in an aeroponic system.

Aeroponic systems were used successfully for studying root growth patterns non-destructively [23], mainly because the whole root system can be easily observed and manipulated. A complimentary, non-destructive approach for measuring root-system size is measuring root capacitance [24]. The main advantages of using root capacitance measurements are the simplicity and rapidity of the procedure, along with the minimal equipment needed for determination of root size. A good correlation was reported between root capacitance and root mass, mostly in hydroponic or sand-based growth media [25]. We found no record of the evaluation of root capacitance as a research tool in aeroponic systems.

The bell pepper is the main crop grown in the Arava valley, Israel, an area characterized by extreme desert conditions [19]. Pepper cultivation covers over 2000 ha [26] and 60% are exported, mainly to the EU and Russia [27]. In this dry area, the main (sometimes sole) source of water used by farmers is saline water [28], in spite of its generally negative impact [17]. Average production for this agro-system typically exceeds 100 ton ha^−1^ [18]. However, frequently, production exhibits large fluctuations, causing economic losses [29]. The aim of this research was to determine whether, and to what extent, the fruit load and water salinity affect the root development of the bell pepper. We hypothesized that an investigation of root growth dynamics’ affected by environmental factors would assist in the development of improved management practices that should prevent seasonal yield fluctuations. Additionally, this study utilized electrical capacitance as a method for the nondestructive, in situ measurement of the root-system-size of aeroponically grown bell pepper plants successfully for the first time.

## 2. Results

### 2.1. Plant Growth Dynamic

**Figure 1:**
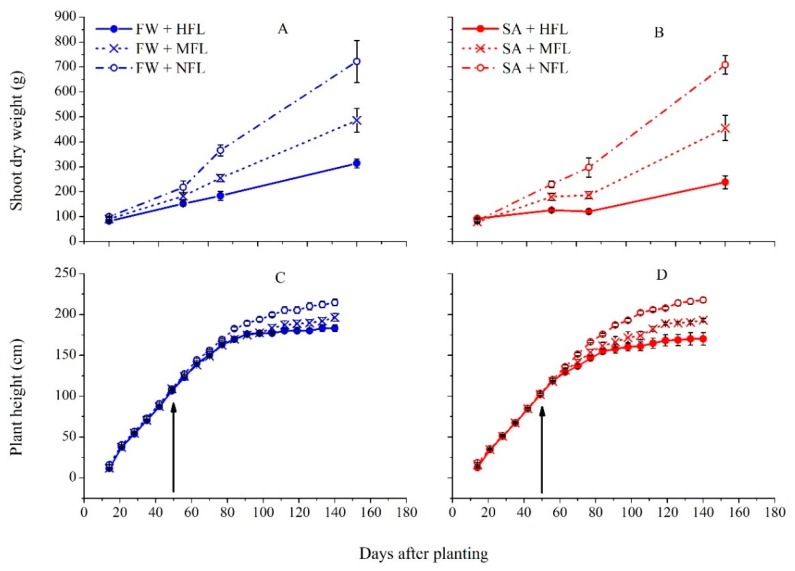
The effects of water quality and fruit load on the plant shoot mass (**A**,**B**) and plant height (**C**,**D**). Bars represent standard errors (±SE) of the means (*n* = 4). The arrows indicate the beginning of first fruit thinning. HFL—high fruit load (solid); MFL—medium fruit load (x); and NFL—no fruit load (empty circle).

Plant growth was assessed by weekly height measurement and destructive biomass sampling (Figure 1). In the plants irrigated with freshwater (FW), fruit load had no effect in the first 50 days after planting (DAP), while at 65 DAP, plant biomass was larger in no fruit load (NFL) and lowest in the high fruit load (HFL) treatment. The effect of fruit load on plant height was evident only 70–80 DAP. In saline (SA) treatment, results followed the same pattern, but the effect of fruit load was more pronounced and the differences were observed at an earlier date (Figure 1B,D). Both plant height and biomass were significantly lower in the SA treatment (Table S1).

### 2.2. Fruit Production

**Figure 2:**
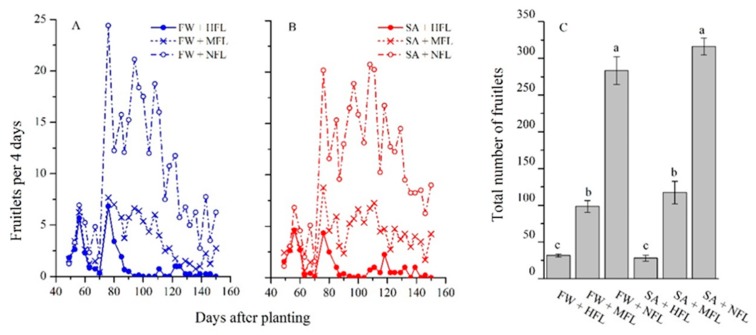
Dynamics of recently formed fruitlets in response to thinning treatments of plants grown in freshwater (**A**) and saline water (**B**), and cumulative fruitlets during the experiment (**C**). HFL—high fruit load (solid); MFL—medium fruit load (x); and NFL—no fruits (empty circle). Bars represent standard errors (±SE) of the means (*n* = 4). Different letters indicate significant difference (*p* < 0.05, two-way ANOVA).

The number of fruitlets produced was affected by the fruit-thinning practice but not by salinity (Figure 2). For all treatments, the first fruitlets were observed around 50 DAP. Later. sharp oscillations in fruit setting were observed, with a pronounced reduction from 60 to 70 DAP (Figure 2A,B). Interestingly, this reduction was noted also in the NFL treatment. Plants under the HFL treatment continued to produce fruitlets from 50 until 90 DAP, and then the rate decreased to a minimum of one or two per four days. The medium fruit load (MFL) plants had significantly higher fruit set number than HFL plants (Figure 2C) and produced fruits at a rate of about five fruitlets per four days until 110 DAP, decreasing afterwards. NFL plants had the highest numbers of fruitlets from 70 DAP.

### 2.3. Root Capacitance

**Figure 3:**
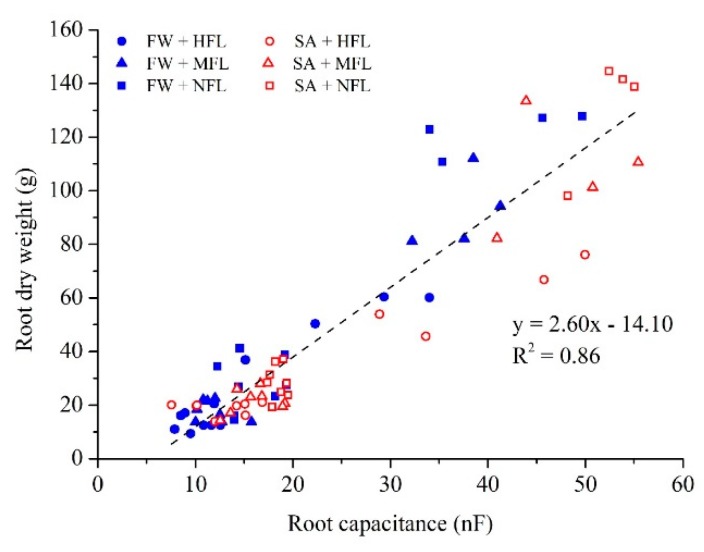
The linear regression of root capacitance and root dry biomass of the pepper plants grown in an aeroponic system and irrigated with fresh water (FW, blue) or saline water (SA, red). HFL—high fruit load; MFL—medium fruit load; and NFL—no fruits.

Root capacitance was monitored weekly and before each destructive sampling. The results of root capacitance readings versus root dry weight are shown in Figure 3. Strong linear correlation was obtained for both FW and SA grown plants (r^2^ = 0.86, *p* < 0.0001). The regression coefficient between root capacitance and weight in the FW treatment was higher compared to those in SA treatment (not shown). However, this difference was not statistically significant (*t*-test, *t* (68) = 2.23, *p* = 0.98), so all measurements were combined to form a single regression equation.

### 2.4. Root Biomass

**Figure 4:**
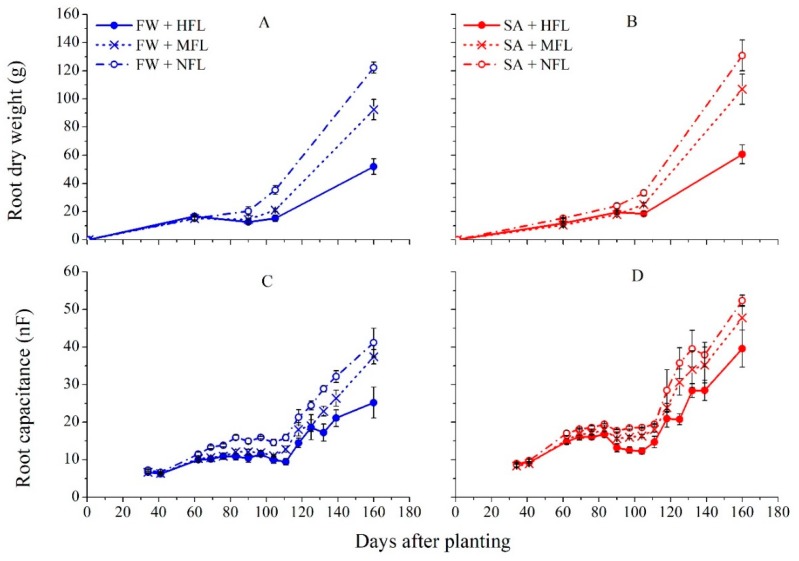
The effects of water quality (fresh water, FW, and saline water, SA) and fruit load on plant root biomass, as measured by direct destructive sampling (**A**,**B**) or converted from root capacitance measurements following the equation presented in Figure 3 (**C**,**D**). Bars represent averages ± SE of four plants. HFL—high fruit load (solid); MFL—medium fruit load (x); and NFL—no fruits (empty circle).

Root growth showed a double sigmoid growth dynamic, being fast during the early vegetative stage (0–60 DAP), followed by a lag period with minimal growth during the main fruit set period (60–90 DAP) and finally, fast increase during fruit ripening and harvest (110–170 DAP, Figure 4).

### 2.5. Root Weight

Root dry mass was significantly affected by time, water salinity and fruit load treatments (Table S1). For the first and second sampling; 60 and 90 DAP, root biomass was not significantly affected by fruit load (Figure 4). At 105 DAP, a gap between the extreme fruit loads was noted and by the end of the experiment, significant differences were found among the three fruit load levels. At 60 DAP, FW treatments had significantly higher root biomasses than SA. From 90 DAP to the end of the experiment, plants irrigated with SA had a significantly higher root mass than those treated with FW (three-way ANOVA, *p* < 0.05, Table S1).

### 2.6. Growth Dynamic by Root Capacitance

The root growth pattern revealed by root capacitance showed vigorous growth at the early stage, until 60 DAP, when a plateau until 110 DAP occurred, and again, rapid root development to the end of the experiment. At the last stage, root development was affected by fruit load. Both destructive and non-destructive methods showed similar trends of root development (Figure 4C,D).

### 2.7. Root/Shoot Ratio

**Figure 5:**
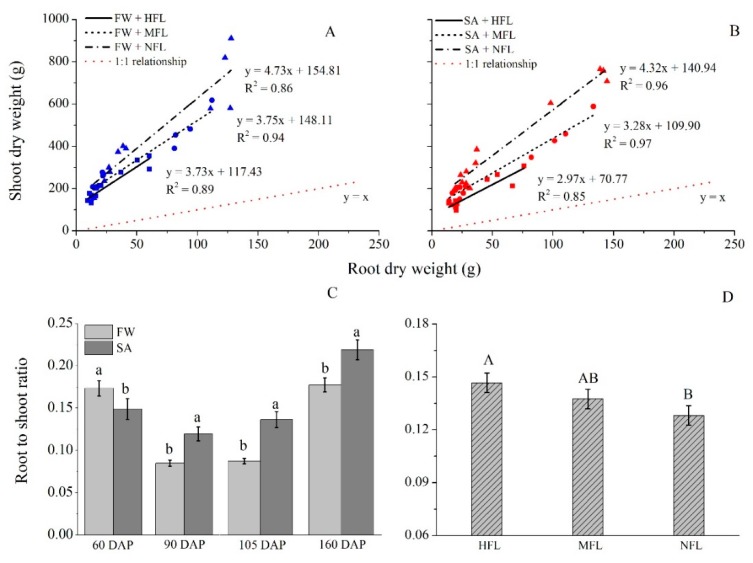
Shoot (fruits not included) versus root biomass regression at two water qualities: fresh water (**A**) and saline water (**B**), and root/shoot ratio during plant development, as affected by water quality (**C**) and fruit load (**D**). Bars represent standard errors of the means (one-way ANOVA, *n* = 12 for C, and ANCOA, *n* = 16 for D). Different letters indicate significant differences (Tukey–HSD test).

Figure 5 provides a graphic illustration of the effect of fruit load and salinity level on root/shoot biomass allocation. In all fruit load treatments, shoot biomass was larger than that of the root. The slope of the linear relationship increased when fruit load decreased. In SA treatments, the slope between shoot and root biomass increased from 3.0 to 3.3 and 4.3 as fruit load decreased from HFL to MFL and to NFL. Table 1 presents the effects of root biomass and fruit load, and their interaction on shoot biomass. The results indicate the slopes are significantly affected by fruit load. The regressions between shoot and root biomass differ among the fruit loads. In the freshwater treatment, however, fruit load did not significantly affect root to shoot correlation (Table 1). The regression coefficient increased from 3.7 for HFL to 4.7 for NFL (Figure 5A). The increase in the regression coefficient indicates that shoot biomass was positively affected when the fruit load was smaller (Figure 5D).

Figure 5C illustrates the root/shoot ratio in response to water quality treatments (regardless of fruit load). Water quality, time and interaction had a significant effect on root/shoot ratio (Table S2). At early plant developmental stages (60 DAP), FW treated plants had a higher root/shoot ratio compared to SA, whereas later in the season (from 90 DAP onwards), SA treated plants had a higher root/shoot ratio (Figure 5C). Regardless of salinity level, fruit load was positively associated with root to shoot ratio being highest in HFL treatment and lowest in NFL (Figure 5D).

### 2.8. Mineral Analysis

Fruit load had minor effect on shoot mineral composition, and therefore, in Table 2 the three fruit load levels were combined. Salinity had a significant, but inconsistent, effect on the mineral composition in shoots and fruits. In the shoots, the main macro-nutrients—N, P and K—were generally higher in the FW treatments. For N, the plant irrigated with FW had higher levels from 90 DAP on, but they were significantly higher after 105 DAP only. For shoot P, levels were higher in the FW compared to the SA, and differences were significant on 60 and 160 DAP only. Potassium concentration in the shoot of the FW treatment was significantly higher on two of the four dates as well (90 and 160 DAP).

In the fruit, the effect of salinity was less pronounced. Nitrogen and P in fruit were generally higher under FW irrigation (significant only on 105 DAP) while K was not significantly affected by salinity.

## 3. Discussion

Salinity is one of the prevalent abiotic stressors with direct implications on root development, especially for irrigated agro-systems in arid and semi-arid regions [30]. Though extensive studies have tested the bell pepper response to salinity [17,31,32], they mainly focused on the aboveground responses. The current study focuses on root growth dynamics as far as they are influenced by fruit load in aeroponically grown bell pepper plants under two salinity levels.

Bell pepper growth dynamics followed a double sigmoid pattern, being high at the vegetative stage (before 60 DAP) and during fruit ripening and harvest (110 to 160 DAP). For the main period of fruit setting, (60 to 110 DAP) root development was minimal, nearly zero. In the arid Arava region, the window for fruit set is limited by the cold night temperatures starting late November, that restrict pepper development [33]. In this limited period, preference of carbon allocation to shoot over root seemed a good strategy. However, fruit thinning did not considerably change the root growth pattern. It appears that during fruit set, both flowers and fruitlets exhibit a strong sink strength that restricts shoot and root development. Hence, lower root growth rate during fruit set is inherent and might be related to a temporary slowing of cambial activity. Inconsistent fruiting is a major problem in pepper production [34] and was also evident in the current study. Two clear waves of fruit set were observed: the first at 50–60 DAP and the second at 70–90 DAP. Water salinity did not affect this inconsistent fruiting pattern. In theory, we might expect fruit thinning to diminish fruiting waves as a result of restraining competition among sinks [29]. Yet, here we found fruiting fluctuations in the complete fruit removal treatments as well, although their magnitude was lower.

Many studies have examined the effect of fruit load on root development in various crops [6,9]. Our study again provides evidence that fruits are a significant factor determining root growth dynamics. Decreasing sink strength by fruit removal led to increased shoot and root growth. High sink strength was previously found to inhibit root and shoot growth in peppers [35] and other crops [6,9]. In citrus fruit, fruit thinning dramatically increased the fruit growth rate [36]. While Nielsen and Veierskov [11] did not find an effect of fruit load on root to shoot ratio of bell pepper, our results indicate that fruit removal changed the root/shoot balance in favor of the shoot. Several reports indicated that high sink strength led to an increase in stomatal conductance and transpiration [37,38]. The reported increase in stomatal conductance in the presence of strong sink might compensate for the substantial decrease in shoot (leaves and branches) growth compared with root growth.

In this study, root growth exhibited a positive response to water salinity, the exact opposite of shoot growth. Similarly, an increase in root to shoot ratio was also reported for soil-grown peppers [39] and poplar trees exposed to mild salt stress [40]. Conversely, most studies reported inhibition in root development in response to salt stress (review: [30]). Such differences in response to salinity can stem from the level of stress experienced by the roots. In our study, moderate stress was imposed and continuous salt leaching was applied. Our results correspond to those of Munns and Tester [41], who reported that roots are less sensitive to salinity than shoots, leading to increase in root to shoot ratio under stress. Munns [14] found that roots can recover faster from salt stress, as found here. Root to shoot ratio was higher in the FW treatment at the early stage, but from 90 DAP onwards, plants irrigated with saline water had higher root to shoot ratios. This indicates that salinity induces modification of assimilate distribution patterns, shifting more assimilates from the shoot to the root. Higher investment of carbon in the root system in response to salinity might be needed to meet the energetic costs of osmotic adjustment and salt exclusion [42], and to enhance the surface area of the root system to compensate for the lower availability of the water. That said, the mechanism of root growth stimulation under low salinity is still unclear [41].

We found that in spite of the extensive root system of the plant irrigated with saline water, their P and K uptake were significantly reduced. The reduction was more pronounced in the shoot than in the fruit, indicating that fruits are a stronger sink for nutrients. Previous studies showed that salinity disturbs efficient nutrient acquisitions [43]. Phosphate versus chloride antagonism was suggested as the mechanism in the roots [44]. Comparable results were found for K, being lower in the shoot of plant receiving higher salinity. Due to the similarities in physicochemical properties, K versus Na antagonism is well established [45] and explains the reduction in K concentration under saline water irrigation. Hence, in our study, ion antagonism rather than inhibition of root development was the probable cause for lower nutrient uptake under salinity.

Finally, this study clearly demonstrates for the first time that root capacitance is a practical, non-destructive method for measuring the root development in aeroponic systems. While characterization of an aboveground plant canopy can be achieved by rapid and simple means, monitoring root development is often tedious, complex and/or destructive. Therefore, using fast and non-destructive method of root capacitance is highly promising [46], and it was shown to be an effective tool for monitoring root development. See Postic and Doussan [47] and many references within. However, some of the challenges limiting broad implementation of root capacitance measurements are the environmental factors that affect the capacitance, mostly moisture content and soil electrical properties [48], often resulting in poor correlations between measured capacitance and root size in real soil conditions [25]. In aeroponic systems, however, a homogenous environment can be achieved by carefully selecting the timing of fogging. Here, we demonstrate that various sink strength and two levels of water salinity do not significantly affect the correlation between root capacitance and root biomass. We found a frequency of 10 kHz was optimal in this specific setup. In various soils, Postic and Doussan [47] found optimal frequency to be two orders of magnitude lower (≈100 Hz) and best predictions of root biomass were found in hydroponics, indicating the importance of environmental homogeniety. The good correlation measured by us (r^2^ 0.86) opens new opportunities for continuous root growth monitoring in aeroponic systems. Recent technological developments are making the aeroponic systems a simple and robust platform that can be used for root development dynamics. That said, further research is needed to establish these findings for various crops and environmental conditions that were out of the scope of the current study.

## 4. Methods

### 4.1. Experimental Design and Growth Conditions

**Figure 6:**
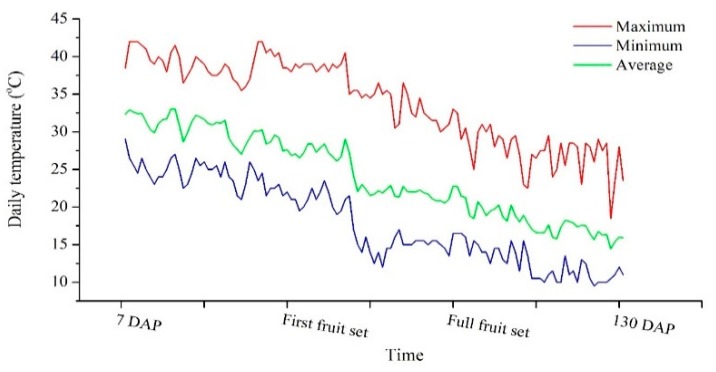
Maximum, minimum and average temperatures in the greenhouse during the experiment.

The experiment was conducted from August 2018 to February 2019 in a controlled commercial greenhouse at the Yair experimental station (Arava R&D Center, Hazeva, Israel; 30°46′ N, 35°14′ E, 130 m below sea level). The greenhouse was equipped with fan cooling, and during the experiment a buffer row of pepper was cultivated around the aeroponic system to reduce border effect. The Arava valley is characterized by clear, hot days and cool nights during the growing season. Temperatures in the greenhouse are presented in Figure 6.

Peppers, *Capsicum anuum* var. 7158 “Canon,” (Syngenta, Gedera, Israel), the most popular variety in the Arava, were grown under two irrigation water salinities and three fruit-loads. Irrigation water was either saline water (SA, EC 2.5 dS m^−1^) originating from a local well or freshwater (FW, EC 0.7 dS m^−1^), provided by the local water supplier. Fertilization was supplied by mixing liquid fertilizer (‘Mor 4-2.5-6’ + micronutrients, Israel Chemicals Ltd., Beer-Sheba, Israel) at a concentration of 0.15% in 1500 L tanks used for irrigation. Fertilization increased the EC by ≈1.0 dS m^−1^. Final mineral concentrations were 72, 20 and 90 mg L^−1^ for N, P and K respectively. Water was monitored weekly for EC, pH and nitrate concentration. If pH dropped below 6.0, sodium carbonate was added to elevate pH to ≈7. Fertilizer was added when nitrate concentration dropped below 60 mg L^−1^, and water and fertilizer were added periodically (depending on the water uptake). When EC increased over 1 dS m^−1^ from its initial value, the whole tank was refreshed (water and fertilizer).

### 4.2. Treatments

Three fruit load levels were applied by manually thinning the fruitlets twice a week: (i) removal of all fruitlets (no fruit load, NFL); (ii) leaving seven fruits per plant and removal of the remaining fruitlets (medium fruit load, MFL); and (iii) no thinning (high fruit load, HFL). The factorial experimental design included: two levels of irrigation water quality (FW and SA) and three levels of fruit load. The total of six treatments were repeated in four containers containing four plants each.

### 4.3. Aeroponic System

**Figure 7:**
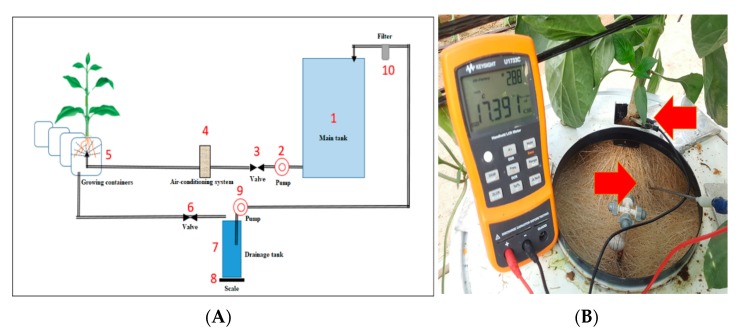
(**A**) Illustration of the aeroponic system used in the current experiment, and (**B**) root capacitance measurement setup in the aeroponic container. Arrows point to the negative and positive electrodes.

The aeroponic system was positioned in the center of the greenhouse surrounded by soil grown peppers. Illustration of the system is presented in Figure 7 and Figure S1. A 1500 L tank (1) containing the water and fertilizer solution served as the source for each type of water, SA or FW. Solution from the main tanks was pumped (2) through temperature regulation system (4) that kept the solution temperature at 21 °C. The solution was sprayed into the plant containers (5) every 4 min for 2 min through a CoolNet sprinkler (Netafim Ltd., Hazerim, Israel) controlled by a valve (3). Drainage from the containers was run through an electronic valve (6) into drainage tanks (7) placed on a scale (8). Next, the water was pumped (9) through a filter (10) back to the main tank.

Two additional containers placed at the south end of each line served as border plants. The 26 containers were arranged in two rows, south–north. Each container holding four plants was 44 cm in diameter. The distance between containers was 40 cm.

Plants were planted on August 22nd, and pest management, plant training and support followed the “Spanish” trellising system with lateral horizontal strings for vertical canopy support, as commonly applied by the local practices [15]. The first fruitlets were observed on October 5th (45 days after planting, DAP), and thinning started on October 9th (49 DAP). One plant from each container was removed 60 (October 22nd), 90 (November 22th) 105 (December 7th) and 160 DAP (February 1st), corresponding to beginning of fruit set, full fruit set, fruit ripening and termination of the experiment. Root, shoot and fruits were separated, dried at 70 °C, weighed and ground to powder that was used for determination of mineral content, as described below.

### 4.4. Measurements

#### 4.4.1. Height

Plant height was measured weekly, starting two weeks after planting, using measuring tape. All plants were measured (i.e., 16 per treatment and number decreases by four after each destructive sampling round).

#### 4.4.2. Fruit Set

The number of fruitlets (fruit diameter >1 cm) was counted twice a week from the beginning of fruit set (49 DAP) throughout the experiment. In HFL and MFL treatments, fruitlets were marked every time. In the MFL treatment, seven fruits of standard size were selected, about half from a full bearing plant. All the other fruits were removed.

#### 4.4.3. Root Capacitance

A handheld LCR meter (U1700, Keysight Technologies, Santa Rosa, CA, USA) was used to measure the root capacitance in nF. Measurements were taken once a week, starting one month after planting until the end of the experiment. Before initiation of the experiment, several preliminary tests were conducted to develop the measurement protocol. Electrode position, measuring frequency and root moisture were examined and optimized. The positive electrode was installed into the root system at angle of 45° above the horizontal, while the negative electrode was clamped to the base of the plant shoot (Figure 7). The root system was sprayed for 2 min and allowed to drain for exactly one minute before taking the measurement, to maintain comparable root moisture for all measurements. The meter was set at 10 kHz. On the day of each destructive sampling, the root capacitance was measured for comparison of the two methods.

#### 4.4.4. Mineral Analysis

Sodium, K and P in shoot and root samples were determined after digestion in a microwave (MARS 6, CEM Corporation, Matthews, NC, USA). Briefly: 500 mg of shoot or fruit was weighed into the digestion tube and subjected to element analyzed using ICP-OES (ICP-OES 5100, Agilent Technologies Inc., USA). Concentration of N was determined using the Dumas combustion method in an automatic elemental analyzer (Rapid N exceed, Elementar, Langenselbold, Germany).

### 4.5. Data Analysis

The JMP (ver.14.0) statistical package (SAS Institute) was used for statistical analyses. Three-way ANOVA was employed to test the effects of fruit load and water quality on root and shoot development. The Tukey–HSD test was used to compare means where significant differences were found; *p* < 0.05 was accepted as the minimum level of significance. The effect of water salinity and fruit load on total fruitlet number was tested by two-way ANOVA. The correlation between root capacitance to root biomass and root to shoot weight were tested by linear regression. The effects of fruit load on recent fruitlet production and on root to shoot ratio were tested using an ANCOVA model wherein fruit load was the independent variable and time was the covariant.

## 5. Conclusions

In the current study, we demonstrated that both irrigation water salinity and fruit sink strength are major determinants for the root growth dynamics of bell pepper plants. Under strong sink strength, shoot development is more restricted than root, leading to the alteration of root to shoot ratio. Additionally, we demonstrated that root capacitance is a valid approach for the non-destructive measurement of root development in aeroponic systems.

## Figures and Tables

**Table 1:** Statistical analysis of root to shoot correlations affected by fruit load level and interaction in the freshwater and saline water independently.

Root to Shoot Ratio	Freshwater		Saline Water	
	*df*	*p*	*df*	*p*
Fruit load	2	0.1761	2	<0.0001
Root biomass	1	<0.0001	1	<0.0001
Interaction	2	0.2865	2	0.0015

**Table 2:** Mineral analysis of shoots and fruit at four growth stages affected by water salinity (*n* = 12). Values are presented as percentages of dry biomasses. Symbols denote significance differences (* 0.05–0.001; ** 0.001–0.001; *** >0.0001; ***n.s.*** not significant).

DAP		Shoot (%)			Fruit		
		N	P	K	N	P	K
	Saline	4.10 ± 0.05	0.41 ± 0.01	6.50 ± 0.12	3.48 ± 0.13	0.59 ± 0.02	4.46 ± 0.24
60	Freshwater	4.03 ± 0.07	0.47 ± 0.01	6.30 ± 0.11	3.60 ± 0.13	0.69 ± 0.04	4.90 ± 0.30
	*significance*	***n.s***	**	***n.s***	***n.s***	***n.s***	***n.s***
	Saline	4.00 ± 0.07	0.33 ± 0.01	5.06 ± 0.08	2.59 ± 0.04	0.39 ± 0.06	3.59 ± 0.10
90	Freshwater	4.20 ± 0.07	0.34 ± 0.01	5.80 ± 0.13	2.66 ± 0.04	0.40 ± 0.05	3.52 ± 0.07
	*significance*	*	***n.s***	***	***n.s***	***n.s***	***n.s***
	Saline	3.57 ± 0.08	0.29 ± 0.01	4.68 ± 0.12	2.23 ± 0.04	0.33 ± 0.01	2.85 ± 0.07
105	Freshwater	3.73 ± 0.08	0.31 ± 0.01	4.97 ± 0.12	2.38 ± 0.09	0.35 ± 0.01	2.90 ± 0.08
	*significance*	***n.s***	***n.s***	***n.s***	**	*	***n.s***
	Saline	3.20 ± 0.05	0.30 ± 0.01	3.16 ± 0.14	3.04 ± 0.15	0.37 ± 0.04	2.80 ± 0.19
160	Freshwater	3.25 ± 0.08	0.34 ± 0.01	3.16 ± 0.14	3.36 ± 0.08	0.42 ± 0.01	3.26 ± 0.14
	*significance*	***n.s***	*	**	***n.s***	***n.s***	***n.s***

## Supplementary Materials

The following are available online at https://www.mdpi.com/2223-7747/9/1/35/s1, Figure S1: Pictures illustrates the aeroponic setup in the commercial greenhouse at the beginning of the experiment (A), close-up on foggers in a single aeroponic container (B) and an overview of the experimental setup at the beginning of the flowering stage (C), Table S1: Factorial three-way ANOVA of shoot biomass, plant height and root biomass as affected by water salinity, fruit load, time and interactions, Table S2: Factorial three-way ANOVA of shoot to root ratio as affected by water salinity, fruit load, time and interactions.

## Abbreviations

FW, freshwater; SA, saline water; HFL, high fruit load; MFL, medium fruit load; NFL, no fruit load; DAP, days after planting.
