# Supplementary material for: Root Development of Bell Pepper (Capsicum annuum L.) as Affected by Water Salinity and Sink Strength
Source: Plants (Basel). 2019 Dec 25;9(1):35. doi: 10.3390/plants9010035 (PMC7020163; doi:10.3390/plants9010035)
Supplement: Supplementary file 1 [file plants-09-00035-s001.pdf]

Supplementary material

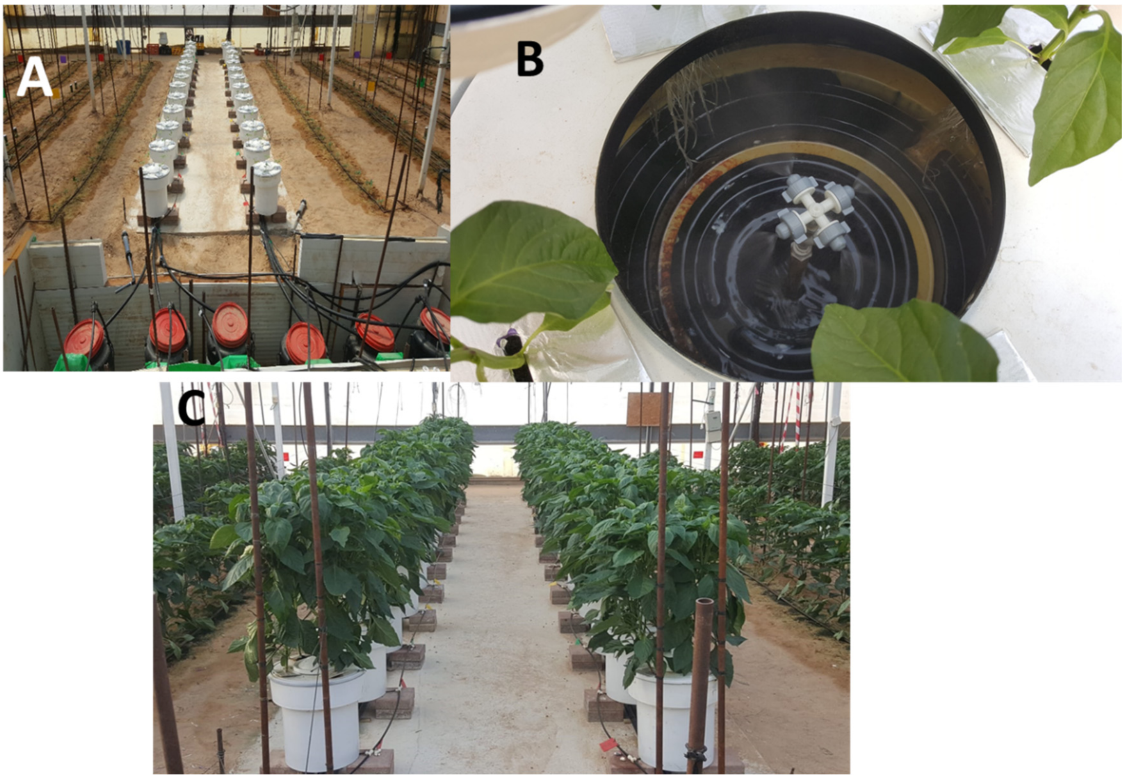

Figure S1: Pictures illustrates the aeroponic setup in the commercial greenhouse at the beginning of the experiment (A), close-up on foggers in a single aeroponic container (B) and an overview of the experimental setup at the beginning of the flowering stage (C)

Table S1: Factorial three-way ANOVA of shoot biomass, plant height and root biomass as affected by water salinity, fruit load, time and interactions.

Factorial Three-way ANOVA	**Shoot DW**			**Plant height**			**Root DW**			**Root Capacitance**		
	df	*F*	*p*	*df*	*F*	*p*	*df*	*F*	*p*	*df*	*F*	*p*
Time	3	233	<0.0001	13	431	<0.0001	3	484	<0.0001	12	115	<0.0001
Water quality	1	6.1	0.0159	1	79.8	<0.0001	1	4	0.0491	1	194	<0.0001
Fruit load	2	80.3	<0.0001	2	303	<0.0001	2	63.3	<0.0001	2	72.8	<0.0001
Time X Water Quality	3	1.64	0.1870.	13	0.81	0.6397	3	2.79	0.0463	12	3.13	0.0004
Time X Fruit load	6	22.4	<0.0001	26	8.35	<0.0001	6	29.4	<0.0001	24	2.16	0.0021
Water quality X Fruit load	2	0.09	0.9096	2	20.2	<0.0001	2	0.09	0.9176	2	0.58	0.5617
Time X Water Quality X Fruit load	6	0.2	0.976	26	0.61	0.932	6	0.28	0.9433	24	0.43	0.9913

Table S2: Factorial three-way ANOVA of shoot to root ratio as affected by water salinity, fruit load, time and interactions

Factorial Three-way ANOVA	***df***	***F***	***p***
Time	3	73.1	< 0.0001
Water quality	1	23.2	<0.0001
Fruit load	2	2.35	0.1024
Time X Water Quality	3	10.4	<0.0001
Time X Fruit load	6	1.98	0.0795
Water quality X Fruit load	2	0.95	0.3915
Time X Water Quality X Fruit load	6	5.75	<0.0001
